# Electric-field-assisted formation of an interfacial double-donor molecule in silicon nano-transistors

**DOI:** 10.1038/srep17377

**Published:** 2015-11-30

**Authors:** Arup Samanta, Daniel Moraru, Takeshi Mizuno, Michiharu Tabe

**Affiliations:** 1 Research Institute of Electronics, Shizuoka University, 3-5-1 Johoku, Hamamatsu 432-8011, Japan

## Abstract

Control of coupling of dopant atoms in silicon nanostructures is a fundamental challenge for dopant-based applications. However, it is difficult to find systems of only a few dopants that can be directly addressed and, therefore, experimental demonstration has not yet been obtained. In this work, we identify pairs of donor atoms in the nano-channel of a silicon field-effect transistor and demonstrate merging of the donor-induced potential wells at the interface by applying vertical electric field. This system can be described as an interfacial double-donor molecule. Single-electron tunneling current is used to probe the modification of the potential well. When merging occurs at the interface, the gate capacitance of the potential well suddenly increases, leading to an abrupt shift of the tunneling current peak to lower gate voltages. This is due to the decrease of the system’s charging energy, as confirmed by Coulomb blockade simulations. These results represent the first experimental observation of electric-field-assisted formation of an interfacial double-donor molecule, opening a pathway for designing functional devices using multiple coupled dopant atoms.

Dopants have played a key role as carrier providers in conventional solid-state electronic devices. In downscaled metal-oxide-semiconductor field-effect transistors (MOSFETs), discrete dopant atoms significantly affect the subthreshold transport characteristics^1^,^2^,^3^. In addition, it was found that dopant atoms can also actively participate in transport as ultra-small quantum dots (QDs) in nano-channel transistors^4^,^5^,^6^,^7^,^8^,^9^,^10^. Dopant atoms are also proposed as part of the basic unit of quantum computing^11^,^12^,^13^,^14^,^15^. For these fundamental applications, coupling of the dopants with each other is an essential factor for obtaining suitable functionalities. Therefore, it becomes critical to identify systems of a few dopant atoms and to control the inter-dopant coupling by external parameters.

The effect of vertical electric field on the control of the electron wave function within a system formed by the potential well of a single dopant atom and a nearby interface has been recently studied both experimentally and theoretically^16^,^17^,^18^,^19^,^20^. However, control of the potential well induced by two dopants at an interface has not yet been demonstrated since identification of such a system in experimental devices remains challenging. In this work, we identify pairs of donors in nano-transistor channels and report, for the first time, the merging of their potential wells at the interface under vertical electric field.

For this purpose, we fabricate and characterize transistors with ultrathin Si channels doped with phosphorus (P) donors. In one type of transistors, the channel was doped with lower doping concentration, for which pairs of donors can be found with high probability. We find that, under the application of a vertical electric field, potential wells induced by two neighboring donors can be merged at the interface, forming an interfacial double-donor molecule-like system. In another type of devices, the channel was selectively doped with higher doping concentration. For this case, we identify clusters of multiple P-donors strongly interacting with each other due to their physical proximity, without assistance of an interface.

## Results

### Ultrathin-channel nano-transistors

**Figure 1:**
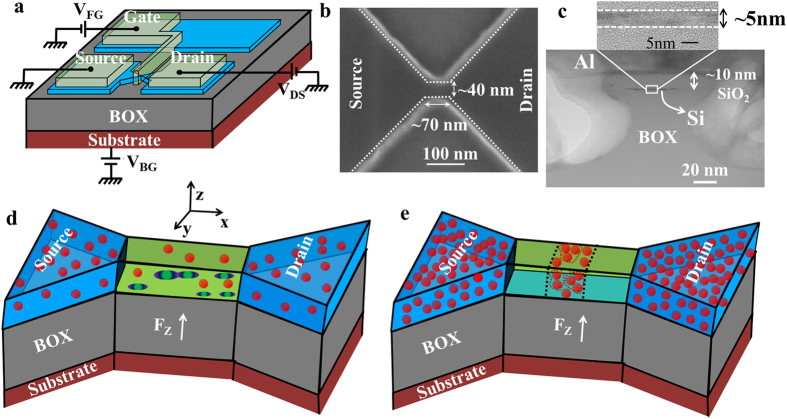
Ultrathin silicon-on-insulator field-effect transistors. (**a**) Schematic structure of an SOI-FET and its measurement setup. (**b**) A SEM image of a typical channel with dimensions below 100 nm. (**c**) TEM image of the channel taken across the channel width. (**d**) Schematic illustration of a possible arrangement of P-donors in a randomly, lower-concentration-doped channel. Under vertical electric field, potential wells can be formed at the interface. Neighboring potential wells may merge forming an interfacial double-donor molecule. (**e**) Schematic illustration of a possible distribution of P-donors in a selectively-doped higher-concentration channel. Clusters of several P-donors are located in the central region of the channel, strongly interacting to form multiple-donor QDs.

A set of devices studied in this work are nano-channel silicon-on-insulator (SOI) MOSFETs, as illustrated in Fig. 1a. Channel length and width were modified as parameters in the ranges of 50–80 nm and 20–50 nm, respectively (details are described in Methods). Figure 1b shows a scanning electron microscope (SEM) image of the channel of one device with final width of ~40 nm and length of ~70 nm. The devices have an ultrathin Si channel (~5 nm), as observed from the cross-sectional transmission electron microscope (TEM) image shown in Fig. 1c. A 10-nm-thick SiO_2_ layer was thermally grown by dry oxidation as gate oxide, on top of which an Al frontgate was formed. The *p*-type Si substrate (boron-doped, *N*_A_ ≈ 1 × 10^16^ cm^−3^) is used as a backgate through a 150-nm-thick buried oxide (BOX) layer. The frontgate and the backgate provide control of the electric field within the Si channel.

The top silicon layer was uniformly doped with P-donors by thermal diffusion from a spin-coated silica film containing P_2_O_3_. Doping concentration was estimated to be *N*_D_ ≈ 1 × 10^18^ cm^−3^ from four-point probe measurements on reference samples. For this concentration, the average distance between neighboring P-donors is ~10 nm. Since the Bohr radius for P-donors in Si (*r*_B_) is ~2.5 nm, the P-donors can be considered as isolated from each other^21^.

Figure 1d schematically shows a possible distribution of P-donors in the channel of one such device. It is also illustrated how each donor induces a potential well at the Si/BOX interface under the application of vertical electric field **F**_z_. The present study mainly focuses on the effect of such electric field on the potential well formed at the interface by a pair of neighboring P-donors.

We have also fabricated another type of SOI-MOSFETs in which the central region of the channel was selectively doped with higher concentration (*N*_D_ > 1 × 10^19^ cm^−3^). For such high concentration, selective doping design is necessary to preserve tunnel barriers in the channel so that tunneling conduction can still be observed without the formation of a highly-conductive channel, like in the case of junctionless transistors. Figure 1e shows a larger number of P-donors in the central region of the channel, forming clusters of strongly-coupled P-donors. For realizing such selectively-doped profile, a 50-nm-wide slit was opened in a doping-mask oxide layer, ~80 nm away from source and drain leads. Lateral diffusion of dopants was suppressed by minimizing the thermal budget during the only subsequent thermal process for gate-oxide formation (see Methods). In these devices, differently from the FETs doped with lower concentration, molecular systems are formed by clusters of strongly-coupled P-donors physically located close to each other^22^, without assistance of an interface.

### Effect of vertical electric field on low-temperature I-V characteristics

**Figure 2:**
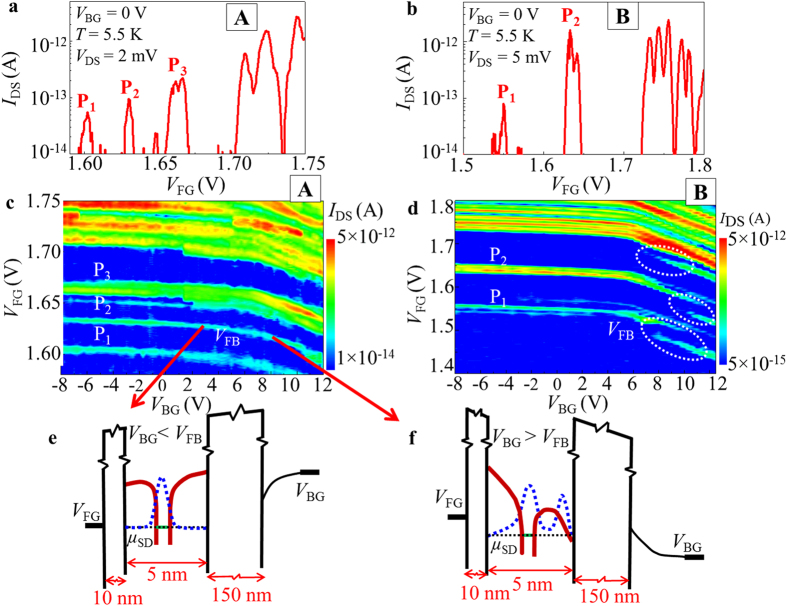
Vertical electric field effect. (**a**,**b**) *I*_DS_–*V*_FG_ characteristics (*V*_DS_ = 2 mV and 5 mV, respectively; *T* = 5.5 K) measured for two different SOI-MOSFETs with channels randomly doped (*N*_D_ ≈ 1 × 10^18^ cm^−3^). Noise level in these measurements in ~10 fA (shown as cut off level for the vertical axes). (**c**,**d**) Contour plots of *I*_DS_ as a function of backgate voltage (*V*_BG_) and frontgate voltage (*V*_FG_) for devices A and B, respectively. Device A shows relatively smooth current traces, while device B shows sudden changes in the current peak positions at positive *V*_BG_. (**e**,**f**) Illustrations of the energy band diagrams and of the electron wave functions at electric fields corresponding to below and above the flatband condition. μ_SD_ is source-drain chemical potential.

For several lower-concentration devices, source-drain current vs front gate voltage (*I*_DS_-*V*_FG_) characteristics were measured at low temperature (*T* = 5.5 K) with backgate voltage *(V*_BG_) as a parameter. In order to observe the effect of the electric field on transport through ground-states only, we map the current peaks only in the low-bias (low-*V*_DS_) region. At higher biases (higher *V*_DS_), more complex behavior is likely to be observed as transport could occur through multiple interactive dopants. Due to the random distribution of dopants, different behaviors are observed for different devices as a function of electric field. In this report, we present two significantly different cases among our observations. Figures 2a-2b show the *I*_DS_-*V*_FG_ characteristics at *V*_BG_ = 0 V for devices labeled A and B, respectively. Several isolated current peaks can be observed before the onset of higher current. These current peaks have irregular intensities (*I*_DS_) and do not exhibit any periodicity, which excludes the possibility of a single multiple-electron QD as their origin. The stability diagrams (plots of *I*_DS_ in the *V*_FG_-*V*_DS_ plane) for each device are also shown in Supplementary Fig. S1a and S1b. Differential conductance was also numerically extracted from this data and displayed as stability diagrams in Supplementary Fig. S1c and S1d. From these diagrams, Coulomb diamonds can be identified confirming that, at least for low-*V*_DS_, transport occurs by single-electron tunneling via a single QD, i.e., most likely the ground state of a donor. At higher *V*_DS_, more complex structures can be observed in the stability diagrams. Based on the quantitative analysis of these diagrams in the low-*V*_DS_ region, we extracted typical parameters for each QD, such as the lever-arm factor, α, and the lateral position along the channel. The results of this analysis are shown in Supplementary Table S1. According to these results, we can conclude that the most reasonable interpretation of our data is for each current peak to be ascribed to single-electron tunneling transport through different individual P-donors acting as QDs, as also described in other works^7^,^8^,^16^. The origin of the complex structures in the stability diagrams at high-*V*_DS_ can be explained based on transport through multiple dopants. The dynamical modification of the potential profile in such multiple-dopant system with the variation in bias and gate voltages could change the equivalent circuit from a single, isolated dopant to interactive, multiple dopants with increasing bias voltage (*V*_DS_). Such modification of the potential profile in a multiple-dopant system has been recently reported based on the potential mapping of doped Si nano-channels by Kelvin probe force microscope (KPFM)^23^,^24^. The change of the transport path from single dopant in the low-*V*_DS_ region to a multiple-dopant system in the high-*V*_DS_ region can explain the occurrence of the complex features in the stability diagram at higher biases. Detailed explanations of the stability diagrams are presented in the Supplementary Information file in Fig. S2. However, in the core part of this work, we avoid such complex configurations by focusing the measurement and analysis in the low-*V*_DS_ region only.

For device A, three isolated peaks (labeled as P_1_–P_3_) can be observed in Fig. 2a at the lowest *V*_FG_’s (1.6 V, 1.63 V and 1.66 V, respectively). Higher-order current peaks appear near the onset of diffusion current for V_FG_ > 1.7 V. Similarly, for device B, two isolated current peaks (labeled as P_1_ and P_2_) can be seen in Fig. 2b at the lowest *V*_FG_’s (1.55 V and 1.64 V, respectively), followed by more complex current peaks for V_FG_ > 1.72 V. The isolated current peaks at the lowest *V*_FG_’s can be ascribed to single-electron tunneling via P-donors with deeper ground-state energies^8^. Current peaks appearing at higher *V*_FG_ can be ascribed to P-donors with shallower ground states. In addition to the main current peaks, faint current traces can be observed, such as seen for device B in Fig. 2b on the left side of the main peak, P_1_. This faint trace is ascribed to tunneling transport through another P-donor energetically close to donor P_1_. The lower intensity of this current trace is most likely due to higher tunnel resistances for this satellite P-donor. These two donors are also likely located close to each other in the channel, forming an intriguing double-donor system that can be directly addressed by electrical measurements. We study the effect of a vertical electric field on the properties of this double-donor system working as a transport-QD for single-electron tunneling.

Vertical electric field can be changed by the two gate voltages, *V*_FG_ and *V*_BG_. Contour plots of *I*_DS_ as a function of *V*_FG_ and *V*_BG_ are plotted in Fig. 2c for device A and in Fig. 2d for device B. In these diagrams, current peaks appear as higher-contrast traces. For both devices, for *V*_BG_ < 5 V each current peak only weakly depends on *V*_BG_. However, for *V*_BG_ ≥ 5 V the current peaks depend more significantly on *V*_BG_. The transition point between these two regimes can be ascribed to the flatband voltage (*V*_FB_) for which the BOX-substrate junction changes from depletion into accumulation mode^25^. The slopes of these current traces in the *V*_FG_-*V*_BG_ plane are dictated mainly by the relative coupling of the transport-QDs to the front gate and to the back gate (*C*_FG_ and *C*_BG_, respectively). For thin Si channel, as in our devices (~5 nm), *C*_FG_ and *C*_BG_ are dominated by the thickness of the gate oxide and buried oxide only. Therefore, these current traces appear as almost parallel to each other in the *V*_FG_-*V*_BG_ plane, even though the QDs have different positions in the channel (as suggested from the analysis of the different slopes of the diamonds in the stability diagrams, shown in the Supplementary Information). This type of behavior is commonly observed for all measured devices. Figures 2e-2f illustrate the band diagrams for *V*_BG_ below and above the flatband voltage. Above flatband voltage, because backgate becomes more strongly coupled to the channel, an increase in *V*_BG_ induces a considerable potential drop across the top Si layer and, implicitly, across donor-induced potential wells found in the channel.

Under the application of this vertical electric field, the electron wave function moves away from the donor site toward the back interface^18^,^26^. During this transition, the system will pass through a condition in which the electron wave function is hybridized between the donor and the interface. The electric field required for such a transition is usually called critical electric field (*F*_C_)^19^,^27^. If the donor is far from the interface, the required *F*_C_ is relatively small and the transition occurs by a non-adiabatic tunneling process. On the other hand, if the donor is close to the interface, *F*_C_ is larger and the transition process is adiabatic. For our measurements, the positive range of *V*_BG_ covers a range of electric fields of 0-~50 mV/nm. Under such electric fields, transport is expected to occur via hybridized donor-interface QDs if the P-donors are close to the interface. This is the case of our devices that have an ultrathin Si channel (~5 nm), as seen in Fig. 1c. In such thin channels, the P-donors are naturally located relatively close to an interface. Figure 2f illustrates the case of large *V*_BG_, for which the electron wave function is extended in the system formed by the donor and the interface well.

For device A, the traces of the current peaks seen in Fig. 2c are smoothly changing as a function of *V*_BG_. However, for device B, some peculiar behavior is observed in Fig. 2d at higher positive values of *V*_BG_ (marked by dashed circles). If we follow the first current peak (P_1_) up to *V*_BG_ > ~8.0 V, it is possible to observe a new current trace suddenly appearing at lower *V*_FG_. At the same time, the upper current trace still appears to continue for a range of *V*_BG_. A similar observation can be made for the second current peak (P_2_) at V_BG_ > ~10.0 V and for the third current peak (not marked) at V_BG_ > ~8.0 V. The estimated electric field at which these current shifts appear is in the range of 20–40 mV/nm. This is the unique phenomenon that we observe in the present study and understanding its origin is the main focus of the following analysis.

The observation of a sudden shift of the current can be most likely ascribed to a sudden increase in the gate capacitance of the transport-QD. At lower *V*_BG_, the transport-QD consists of one P-donor’s potential well. However, as *V*_BG_ is increased, the potential expands at the interface and in the lateral direction^18^ and, as a result, gate capacitance gradually increases. Since another neighboring P-donor also experiences a similar expansion, the potential wells of such two donors will suddenly merge at a critical *V*_BG_. This merged potential well, formed near the interface, will thus become the new transport-QD having a suddenly increased gate capacitance. The system can be treated from this point on as an interfacial double-donor molecule. This model will be treated in more details in the Discussion section by simulations of potential landscapes and Coulomb blockade transport. The Supplementary Fig. S3 shows an additional analysis of the correlation between the shift of the current trace to lower *V*_FG_ and the increase of gate capacitance.

**Figure 3:**
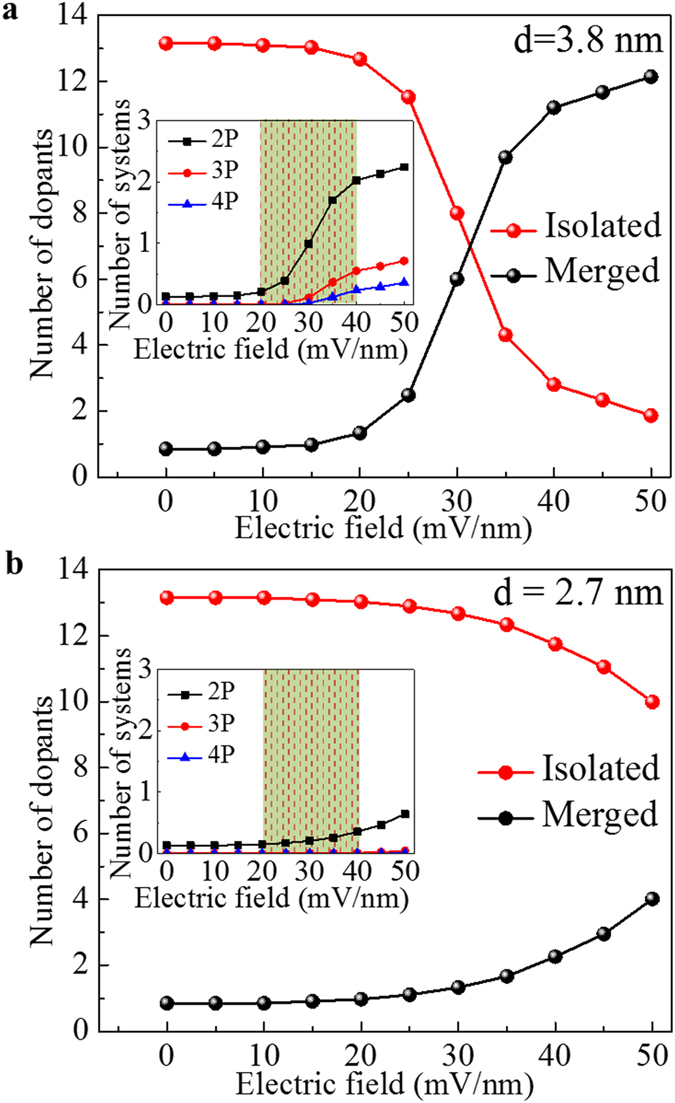
Calculation of probability of donor-well merging. (**a**,**b**) Number of isolated and merged dopant-induced wells calculated probabilistically as a function of electric field for two different distances of P-donors from the back interface: 3.8 nm [(**a**)] and 2.7 nm [(**b**)]. Insets: number of systems in the device channel containing 2, 3, or 4 merged P-donor wells assuming a Poisson distribution of the P-donors. The green zones correspond to the range of electric fields estimated for our experiment.

In order to evaluate the possibility of this occurrence, we first estimate the probability of formation of merged donor-induced wells with increasing vertical electric field. For that purpose, we consider that the radius of lateral confinement for the donor-induced well changes with the electric field as calculated in Ref. 18 by tight-binding simulations. The donor-induced wells are treated as having spherical symmetry for simplicity. Within this model, we calculate the probability of finding at least pairs of overlapped P-donor wells based on the formula given below^28^:


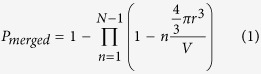


where *N* is the total number of donors in the channel, *r* is the radius of the lateral confinement of the donor-induced well and *V* is the volume of the channel. The total number of donor atoms in the channel is ~14, as calculated from channel dimensions (70 × 40 × 5 nm^3^) and average doping concentration (*N*_D_ = 1 × 10^18^ cm^−3^). After calculating the above probability, we can evaluate the number of donor-wells distributed as isolated and as merged. For instance, assuming that all donor atoms are situated at distance 3.8 nm from the Si/BOX interface, this evaluation leads to the results shown in Fig. 3a. This figure shows that the number of merged donor-wells gradually increases with increasing electric field. At the same time, the number of isolated donor-wells gradually decreases. Systems of merged donor-wells can be further classified as a function of the number of P-donors that are coupled together within a distance smaller than 2*r*_B_ from each other. We calculate the probability of finding systems containing 2, 3, and 4 merged P-donor wells assuming a Poisson distribution of the donors in the lateral plane^29^. The basic result is shown as an inset in Fig. 3a. This result suggests that, for electric field of approximately 20–40 mV/nm (marked as a green zone in the inset), donor-well pairs (double-donor molecule-like systems) can be found in the channel. At the same time, the chance of finding higher-order merging of multiple P-donor wells is negligible.

A similar probability calculation was also carried out for the case of P-donors situated at 2.7 nm from the Si/BOX interface. The results are presented in Fig. 3b. In this case, the probability of formation of merged P-donor wells is significantly reduced. The number of donor-well pairs is practically zero for the range of electric fields corresponding to our measurements, as shown in the inset of Fig. 3b. Therefore, for donors close to the Si/BOX interface, it is unlikely to observe merging of neighboring donor-wells within our experimental conditions.

Based on this probability calculation, it can be concluded that P-donors located closer to the front interface can readily merge their potential wells at the back interface as electric field is increased. On the other hand, if the donors are located closer to the back interface, there is a significantly lower probability to induce merging of their potential wells. This interpretation may be correlated with a specific P-donor arrangement in device B, which may be more favorable for the formation of the interfacial double-donor molecule system.

**Figure 4:**
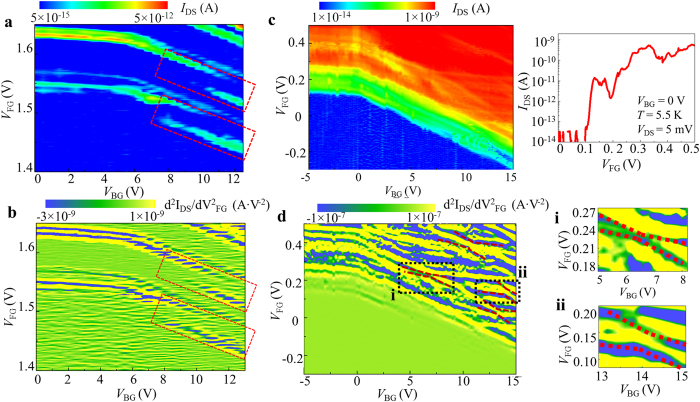
Transport characteristics for devices doped with lower and higher concentration. Contour plots of *I*_DS_ (top panels) and d^2^*I*_DS_/d*V*_FG_^2^ (bottom panels) as a function of backgate voltage (*V*_BG_) and frontgate voltage (*V*_FG_) for two types of devices: (**a**,**b**) randomly doped with lower doping concentration (*N*_D_ ≈ 1 × 10^18^ cm^−3^); (**c**,**d**) selectively-doped with higher doping concentration (*N*_D_ > 1 × 10^19^ cm^−3^). All data is measured at T = 5.5 K and *V*_DS_ = 5 mV. Regions of current shifts are marked by dashed rectangles in (**a**,**b**). *I*_DS_–*V*_FG_ characteristics (*V*_BG_ = 0 V) for the selectively-doped high-concentration FET are shown as a side panel in (**c**). In (**d**), within the complex pattern of fine traces, several anti-crossing structures are marked by dashed lines, suggesting interaction between two QDs. Zoom-in plots of two such regions [marked as (**i**) and (**ii**)] are presented in the right-side panels of (**d**).

Figure 4a shows a zoomed-in *V*_FG_-*V*_BG_ diagram within the positive-*V*_BG_ region, focusing only on the two lowest*-V*_FG_ prominent current peaks. As a way to emphasize the fine features, a second-order derivative of the current as a function of *V*_FG_ is also presented in Fig. 4b. The faint current trace below the main peak becomes more prominent in Fig. 4b. As explained earlier, such fine trace can be ascribed to another P-donor located in the vicinity of the main transport donor. From both figures, it can be clearly observed that the current traces suddenly shift to lower *V*_FG_ in a range of positive *V*_BG_. The regions in which these current shifts occur are marked by dashed rectangles.

More complex interactions are expected for devices with channels selectively-doped with higher concentration. An example for one such device is shown in Fig. 4c by the diagram of *I*_DS_ in the *V*_FG_-*V*_BG_ plane. *I*_DS_-*V*_FG_ characteristics (*V*_BG_ = 0 V) are also shown as the side panel of Fig. 4c. In these figures, current peak envelopes containing a sub-structure of fine features can be observed. These are ascribed to QDs formed by multiple P-donors in the center of the channel by the selective-doping technique. As demonstrated in our previous report^22^, such QDs exhibit a molecule-like behavior due to the strong interaction of multiple P-donors, without assistance of an interface. The fine features can be emphasized by plotting the second-order derivative of *I*_DS_ as a function of *V*_FG_, as illustrated in Fig. 4d. Most importantly, within the complex pattern of traces, it is possible to identify several anti-crossing features as marked in Fig. 4d by dashed lines as eye guides. Several examples are shown also as zoom-in plots in the side panels of Fig. 4d. Such features are usually ascribed to sequential tunneling in systems of two QDs coupled with different strengths to different gates. As fundamental examples, simple anti-crossing features have been recently reported for pairs of acceptors^30^ or pairs of donors^31^,^32^ in Si-transistor channels. In our highly-doped devices, QDs formed by closely-spaced P-donors may interact with each other, giving rise to the observed anti-crossing features. Thus, the experimental observations for these devices illustrate the case of strong coupling of neighboring donors induced by a higher doping concentration. Inter-donor coupling cannot be easily controlled by the electric field in this type of devices.

## Discussion

**Figure 5:**
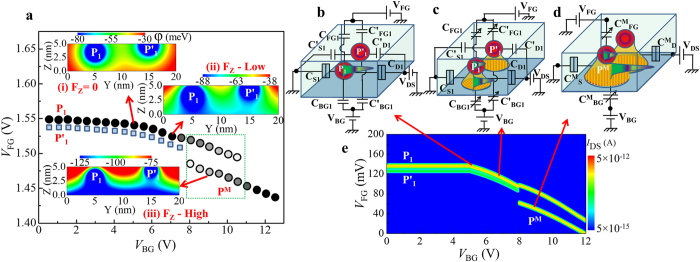
Transport mechanism and Coulomb blockade simulation of merging donor-wells. (**a**) Schematic representation of the evolution of one main current peak (P_1_) with increasing *V*_BG_. P′_1_ corresponds to a fine current trace due to a satellite neighboring P-donor. Insets: potential landscapes of a double-donor system (with donors located at 3.8 nm and 4.5 nm, respectively, from the back interface) for different vertical electric fields (F_z_). Three different regimes are shown: (**i**) F_z_ = 0 mV/nm; (**ii**) F_z_–low; **(iii**) F_z_–high. (**b**) Equivalent circuit of two parallel-coupled donor-induced QDs. **(c)** Equivalent circuit of two parallel-coupled donor-well QDs with increasing gate capacitance due to the gradual expansion of the donor-wells at the interface. (**d**) Equivalent circuit of merged two-donor-well (forming a single QD with larger gate capacitance). (**e**) Simulated *I*_DS_ plotted in the *V*_FG_-*V*_BG_ plane, as obtained for the sequence of equivalent circuits shown in (**b**–**d**).

The main experimental observations for device B with lower doping concentration are schematically summarized in Fig. 5a. The key finding is the sudden shift of the current peak to lower *V*_FG_ as *V*_BG_ is increased. As suggested earlier, this shift could be related to the merging of neighboring P-donor wells, a phenomenon which can occur with relatively high probability in the lower-concentration devices. Within this model, the electron wave function is first gradually shifted from the main transport-donor (P_1_) toward the interface and then it abruptly expands due to merging with a neighboring P-donor (P′_1_) well. This satellite donor is expected to be energetically and spatially close to the main transport-donor, but it gives rise to a lower-intensity current possibly because of higher tunnel resistances.

The system can be illustrated by the electronic potential landscapes induced by the Coulomb potentials of such two P-donors. We consider a case of two donors situated at 3.8 nm and 4.5 nm away from the back interface and separated from each other in the *y* direction at 10 nm. The *y*-*z* cross-sectional potential landscapes for different electric fields are presented as insets in Fig. 5a for three different cases: (i) at zero electric field (F_z_ = 0) ; (ii) at low vertical electric field (F_low_); (iii) at higher vertical electric field (F_high_). For zero-field condition [(i)], the two donor-wells are significantly separated from each other. For a low vertical electric field [(ii)], the potentials of both donors expand towards the interface and slightly in the lateral direction^18^, but they still remain separated from each other. For a large enough electric field [(iii)], due to further lateral expansion, the two donor-induced wells merge with each other at the back interface. This situation, shown in the lower inset, corresponds to the formation of the interfacial double-donor molecule.

Other possible reasons for the appearance of the new current trace at lower *V*_FG_ can be excluded. For instance, a current shift may be intuitively ascribed to trapping of a charge carrier into another P-donor or into an interface trap state. However, since the charge carriers are electrons, trapping of such a negative charge should shift the channel potential to higher values and *V*_FG_ would shift to more positive values. This is inconsistent with the polarity of the experimentally observed shift (towards more negative *V*_FG_). An extended argument is introduced in Supplementary Fig. S4. Another possible model could be that the current shift is due to a sudden transition of the electron wave function from the donor to the interface by a tunneling mechanism. However, within the ultrathin Si channel of our devices, such transition is expected to occur most likely adiabatically, which would not induce any sudden changes in the current peak position. Hence, merging of two neighboring donor-wells remains as the most plausible model.

In order to theoretically explain the shift of the current peak to lower *V*_FG_ when the donor-wells merge with each other, we study tunneling transport based on the Coulomb blockade theory^33^. Within this framework, merging of donor-wells leads to an increase in the QD area and, implicitly, in the gate capacitance of the resulting QD. As a consequence, the charging energy of the system is reduced. When the system has a reduced charging energy, the current peak is expected to appear at lower *V*_FG_.

For clarifying this point, we simulate the system of two donor-wells using a circuit of two parallel QDs, as schematically illustrated in Fig. 5b–d. Simulation details are given in Methods. The QD labeled as P_1_ works as main conduction path and is coupled to source and drain by tunnel junctions with capacitances C_S1_ and C_D1_, respectively. The satellite QD P′_1_ is coupled to source and drain via tunnel junctions with capacitances C′_S1_ and C′_D1_, respectively, but with higher tunnel resistances. The two QDs are coupled to the gates (frontgate and backgate) by non-tunnel capacitors with capacitances (C_FG1_; C_BG1_) and (C′_FG1_; C′_BG1_), respectively.

The simulation parameters are estimated based on the device geometry and the proposed model. In addition, since the donor-well gradually expands at the interface, its gate capacitance is also expected to gradually increase with increasing electric field. This behavior is also incorporated in the present simulation as a *V*_BG_-dependent gate capacitance of the QDs. With this phenomenological model, simulation was carried out by using the successive circuits shown in Fig. 5b–d. The simulated *I*_DS_ is plotted in the *V*_FG_–*V*_BG_ plane in Fig. 5e. The circuit shown in Fig. 5b corresponds to the zero electric-field situation. For such condition, the current trace starts to become more strongly dependent on *V*_BG_. Figure 5c illustrates the increased gate capacitance due to the gradual expansion of the QD as *V*_BG_ is increased. This occurs for *V*_BG_ beyond the flatband voltage, as the electron wave function expands at the back interface. Finally, Fig. 5d illustrates the situation when the two QDs merge to form a single larger QD. This is the most important effect captured by this simulation because it corresponds to the merging of the two neighboring donor-induced potential wells at the interface. From the diagram in Fig. 5e, it can be observed that the current peak suddenly shifts to lower *V*_FG_. This shift, ascribed to the sudden reduction of the system’s charging energy, is consistent with our experimental observation.

Finally, it is expected that two electrons can be accommodated in such a double-donor QD, since each P-donor can accommodate only one electron, except at very low temperatures^6^,^9^,^10^. Thus, two current peaks should be observed as a function of *V*_FG_. Indeed, in Fig. 5e two current peaks can be reproduced for *V*_BG_ larger than a critical value. These two current peaks correspond to the double-electron occupancy of the merged QD (P^M^). This can explain the two current traces experimentally observed and illustrated in Fig. 5a, overlapped for a range of *V*_BG_, as marked by a dotted rectangle. The higher-*V*_FG_ trace appears practically in the continuation of the original single-donor-well trace. This suggests that the potential of the interfacial QD containing one electron is comparable with the potential of the original single-donor well. Based on these results shown in this section, it can be concluded that this Coulomb blockade simulation approach provides qualitative support for the interpretation of our experimental findings. Additional simulation data shown in the Supplementary Fig. S3 confirm the correlation between the shift of the current trace and the gate capacitance increase induced by merging of two single-donor-wells, with a good agreement with our experimental results.

In conclusion, we demonstrated experimentally that two donors in an ultrathin SOI-FET channel can merge their potential wells at an interface by applying vertical electric field. By monitoring the single-electron tunneling current, we observed a transition of the QD from a single-donor well to an interfacial double-donor molecule-like structure. This transition is observed as a sudden shift of the current peak to lower *V*_FG_ due to a reduction in the charging energy of the merged interface well. This result can provide a pathway to control the electron wave function in systems of multiple P-donors connected via an interface, essential for a wide range of dopant-based applications.

## Methods

### Device fabrication and structure

Samples were fabricated from silicon-on-insulator (SOI) wafers, with a 150-nm-thick buried oxide (BOX) layer. All processes have been carried out in a clean room environment, using CMOS-compatible techniques. Initial SOI thickness was 55 nm, but it was thinned down by sacrificial oxidations and usual oxidation processes to a final thickness of ~5 nm (as measured by transmission electron microscope (TEM) images). Channel patterning was done using an electron-beam lithography (EBL) technique, with the channel width set as the main parameter (final channel widths are in the ~20–50 nm range) for uniformly doped low concentration devices. High quality gate oxide is formed by thermal oxidation process with an average thickness of ~10 nm for all devices. However, for the selectively-doped devices, gate oxide was formed by wet thermal oxidation (800 °C, 15 min) in order to minimize lateral thermal diffusion of dopants after selective doping. Standard Al metallization technique is adopted to make the gate, source, and drain contacts. The *p*-type Si substrate (doped with boron (B), with concentration ~1 × 10^16^ cm^−3^) is used as a backgate.

### Measurement setup for electrical characterization

Electrical characteristics were measured with Agilent 4156C and Agilent B1500A precision semiconductor parameter analyzers using a variable-temperature prober station. The samples were placed in a high-vacuum chamber for the *I*-*V* measurements, carried out mainly at low temperatures (~5.5 K). In all measurements, the source electrode was grounded and a small bias was applied to the drain electrode (typically, *V*_D_ = 2–5 mV). The frontgate voltage (*V*_FG_) and backgate voltage (*V*_BG_) were swept as variable parameters.

### Monte Carlo simulations

The theoretical *I*_DS_-*V*_FG_ characteristics are calculated numerically using Monte Carlo simulation^34^, by ignoring co-tunneling effects, as a function of *V*_BG_. According to the orthodox theory of Coulomb blockade, forward and reverse tunneling rates of electrons across a junction are given by:


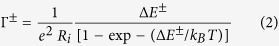


where *e* is an elemental charge and Δ*E*^±^ is the change in total charging energy due to forward and reverse tunneling events. The temperature *T* is set to be 5.0 K for the present study, consistent with our experimental conditions. By considering a suitable range of tunneling parameters based on the experimental situation, *I*_DS_-*V*_FG_ characteristics are simulated in the experimental range of positive *V*_BG_.

## Additional Information

**How to cite this article**: Samanta, A. *et al.* Electric-field-assisted formation of an interfacial double-donor molecule in silicon nano-transistors. *Sci. Rep.* **5**, 17377; doi: 10.1038/srep17377 (2015).

## Supplementary Material

Supplementary Information
