# Supplementary material for: Electric-field-assisted formation of an interfacial double-donor molecule in silicon nano-transistors
Source: Sci Rep. 2015 Nov 30;5:17377. doi: 10.1038/srep17377 (PMC4663623; doi:10.1038/srep17377)
Supplement: Supplementary Information [file srep17377-s1.doc]

Supplementary Information

**Electric-field-assisted formation of an interfacial double-donor molecule**

**in silicon nano-transistors**

Arup Samanta, Daniel Moraru, Takeshi Mizuno & Michiharu Tabe*

*Research Institute of Electronics, Shizuoka University, 3-5-1 Johoku,
Hamamatsu 432-8011, Japan*

**Email: romtabe@rie.shizuoka.ac.jp*

**Stability diagrams for device A and device B**

Stability diagrams (contour plots of *I*_DS_ in the *V*_FG_-*V*_DS_ plane) are useful for identifying the properties of the quantum dots (QDs) responsible for tunneling-transport current peaks. Such stability diagrams, also regularly plotted as differential-conductance plots in the *V*_FG_-*V*_DS_ plane, can provide information about coupling of small geometric QDs or donor-induced QDs to gates and leads in nanoscale single-electron tunneling transistors.^1-3^

Here, in Figs. S1**a** and S1**b**, we show the stability diagrams measured at low temperature (*T*=5.5 K) for the two devices discussed in the manuscript (labeled device A and device B in Fig. 2). Coulomb diamonds (regions of low current, below the noise level in our measurements, ~10 fA) can be observed for both devices. The boundaries of these regions are delineated by solid lines, extended as dashed lines at higher *V*_DS_’s. Differential conductance (numerically extracted from the *I*_DS_-*V*_DS_ data) is also plotted in the *V*_FG_-*V*_DS_ plane in Figs. S1**c** and S1**d** for device A and device B, respectively. These plots allow for a clearer identification of the Coulomb diamonds’ boundaries, in correlation with the *I*_DS_ plots. In these devices, an average of 10-14 dopants is likely to be present in the channel region. Thus, the channels of our devices are effectively multiple-dopant systems. In such a system with multiple-dopant, it could be noted that dopant atoms involved in the transport change from single to multiple (interactive) dopants with increasing the applied bias (*V*_SD_), as explained in details later. In the low-bias region, which is the core part of the Manuscript, we observed simple Coulomb diamonds, without any periodicity, corresponding to successive peaks presented in the Manuscript. Hence, it is natural to assume that different current peaks can be ascribed to single-electron tunneling through different P-donors. However, at higher *V*_DS_, more complicated structures, such as some additional diamonds delineated by dashed lines, can be observed, which makes the overall interpretation of the stability diagrams more complex. Such additional diamonds could be explained based on transport through interactive multiple dopants in the high-*V*_DS_ region only, as described in details later. Nevertheless, these high-*V*_DS_ features are not expected to affect the low-*V*_DS_ region, which are the main focus of our analysis in the Manuscript.


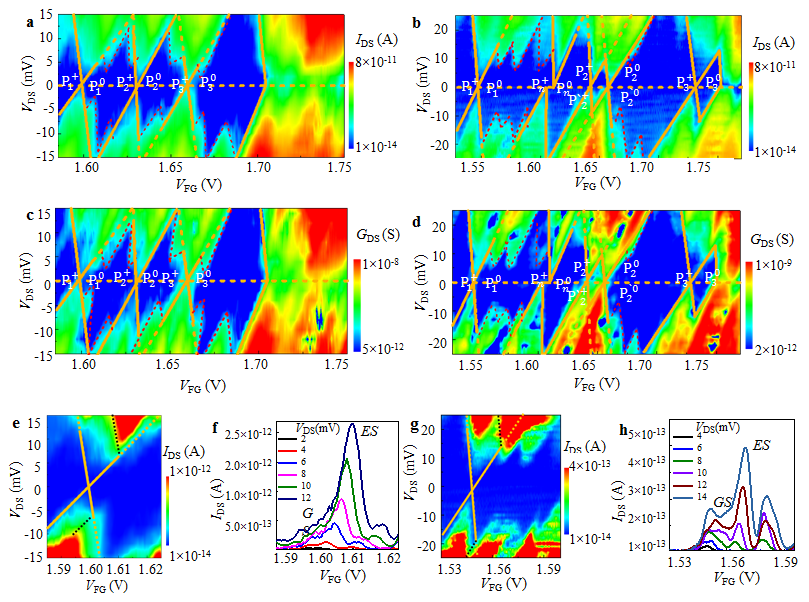


**Supplementary Figure S1 ǀ Stability diagrams for device A and device B.** (**a)-(b)** Stability diagrams (plots of *I*_DS_ in the *V*_FG_-*V*_DS_ space) measured at *T*=5.5 K for device A and device B shown in the manuscript. Solid lines are drawn as boundaries of the zero-current (Coulomb diamond) regions, extended to higher *V*_DS_ values as dashed lines. Different current peak regions are assigned to different P-donors. **(c)-(d)** Conductance stability diagrams (plots of differential conductance *G*_DS_=d*I*_DS_/d*V*_DS_ in the *V*_FG_-*V*_DS_ space) for device A and device B shown in the manuscript. Solid lines are drawn as boundaries of the Coulomb diamonds, extended to higher *V*_DS_ values as dashed lines. Lines are consistently drawn by correlation between *I*_DS_ and *G*_DS_ plots. (**e-f), (g-h)** First current peak region and series of *I*_DS_-*V*_FG_ curves as a function of *V*_DS_ for device A and device B, respectively. In the contour plots, fine steps of current are indicated by dashed lines. Such current steps are more clearly observed in the *I*_DS_-*V*_FG_ traces [labeled as ground state (GS) and excited state (ES)].

In the lower panels of Fig. S1, we show the first current region for each device [as contour plots (Figs. S1**e** and S1**g**) and as series of *I*_DS_-*V*_FG_ curves (Figs. S1**f** and S1**h**)]. For each device, we can identify some fine features or steps (indicated by dashed lines) which can be ascribed to contributions to transport due to an excited state (ES). We evaluate the energy difference between ground state (GS) and ES to be ~10 meV, which is in reasonably good agreement with the typical value of 12 meV known for P-donors in bulk Si. Features appearing at higher *V*_DS_ cannot be easily identified as excited states, since more complex interactions with different P-donors could play a significant role in transport at such high biases.

Now, we explain the overall stability diagram of these devices and the possible origin of low-*V*_DS_ and high-*V*_DS_ features. In the low-bias region, we observed simple Coulomb diamonds for successive peaks, as presented in the manuscript. However, in the high-bias region, we observed a complex diamond structure corresponding to each current peak. Since the channels of our devices contain several dopants (~10-14), it is possible that the potential profile of the system drastically changes from low-bias region to high-bias region and even in different gate voltage regions. Thus, the gradual modification of equivalent circuit with the variation in drain bias and gate voltage may occur, changing the transport system from a single, isolated donor at low biases to an interactive multiple-donor system at higher biases. We recently reported such phenomena observed by the changes in the potential measured on doped nano-channels by Kelvin probe force microscope (KPFM).^4,5^ In these papers, we showed how the potential profile changes with changes of bias and gate voltages. At low bias, mostly transport through individual dopants can be observed. However, at high-bias, a drastic modification in potential profile makes transport through multiple dopants more reasonable to be expected. Thus, we observed a dynamical modification of equivalent circuits from transport through individual dopant to transport through interactive multiple dopants with the change of bias region from low-*V*_DS_ to high-*V*_DS_.

The modification in the transport path from isolated dopant to interactive multiple dopants can happen whenever we go from low-bias to high-bias region in multiple-dopant system. So, the low-bias features can be explained based on transport through individual dopant, while high-bias features can be explained based on transport through multiple dopants. Overall, the stability diagram of ‘Device A’ can be explained based on the model presented in Fig. S2. For that, we consider two different possible equivalent circuits for low-*V*_DS_ and high-*V*_DS_ regions, as presented in Fig. S2b and S2d, respectively. In the low-*V*_DS_ region, for each current peak, an isolated dopant participates in the transport. In Fig. S2a, we show how potential-profile changes as successive electrons are transported; corresponding current peak structure and schematic stability diagram in the low-*V*_DS_ region are also illustrated. However, in the high-*V*_DS_ region, we can expect a more complex transport path, i.e., multiple dopants can participate in transport. So, each current peak may appear as a split peak depending on the number of dopants participating in the sequential tunneling transport.^6^ The modifications of the potential profile with the successive electron transport in the high-*V*_DS_ region for ‘device A’, along with the corresponding current peak structure and stability diagram, are presented in Fig. S2c. This type of modified equivalent circuit in the high-*V*_DS_ region can explain the complex structures observed in the stability diagrams for our multiple-dopant system. Such phenomena are indeed naturally expected in a multiple-dopant system in the high-bias region. In a similar way, we can explain the stability diagram of ‘device B’ with different combination of interactive dopants in the high-*V*_DS_ region. However, we note that the high-bias region is irrelevant for our manuscript scenario. The low-bias current peaks, which represent the main scenario of the manuscript, remain as originated from individual P-donors.


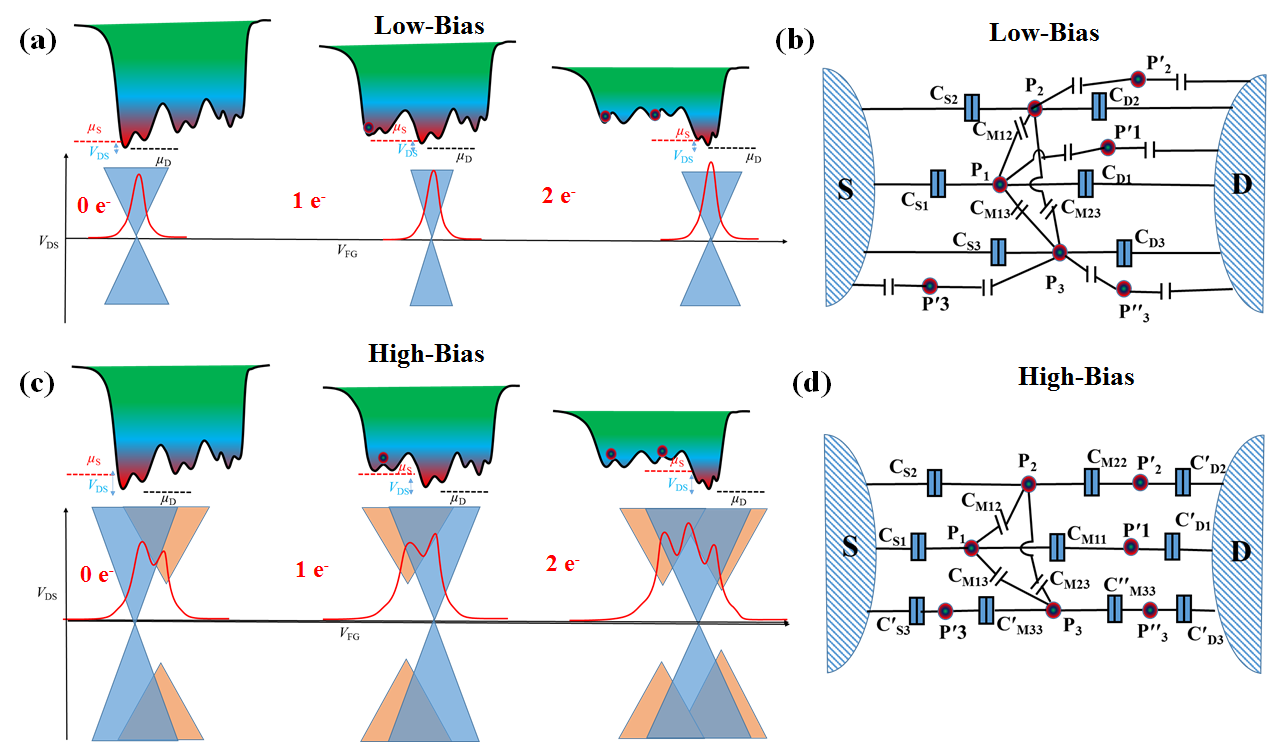


**Supplementary Figure S2 ǀ Bias dependent dynamics of potential profile of the channel containing multiple dopants.** (**a**) Modification of potential profile for successive electron transport along with schematic current peak-structure and stability diagram in the low-*V*_DS_ region. (**b**) Low-bias possible equivalent circuit for ‘device A’. (**c**) Modification of potential profile for successive electron transport along with schematic current peak-structure and stability diagram in the high-*V*_DS_ region. (**d**) High-bias possible equivalent circuit for ‘device A’.

To give additional support for the origin of the low-bias features, we performed a more detailed quantitative analysis of each current peak from the stability diagrams and conductance plots for the low-*V*_DS_ region only. By evaluating the slopes of the Coulomb diamonds corresponding to each current peak (in the low-*V*_DS_ region), we can calculate *α,* the lever-arm factor [*α=C*_G_*/(C*_G_*+C*_S_+*C*_D_*)*, with *C*_S_, *C*_D_, and *C*_G_ being the capacitances between the QD and source, drain, and gate electrodes, respectively]. *α* represents the coupling strength of each QD to the front gate. Calculated values of *α* for each current peak are presented in Tables S1**a** and S1**b** (first column) for device A and device B, respectively. From Table S1**a**, values of α for peaks 1-3 are approximately 0.43, 0.64, and 0.51. From Table S1**b**, α values for the main transport peaks 1-3 are approximately 0.76, 0.67, and 0.53 (two more additional peaks can be identified, also with different values for α of approximately 0.85 for P_n_ and 0.58 for P′_2_). These differences in the values of lever-arm factors are within the order of acceptable range reported for dopant-based system. For example, in reference 2, two quantum dots are distinguished by their apparently small difference in lever-arm factors (0.220 and 0.245). Thus, these results show that each current peak is due to different QDs with different couplings to front gate (different front gate capacitances), relative to total capacitance.


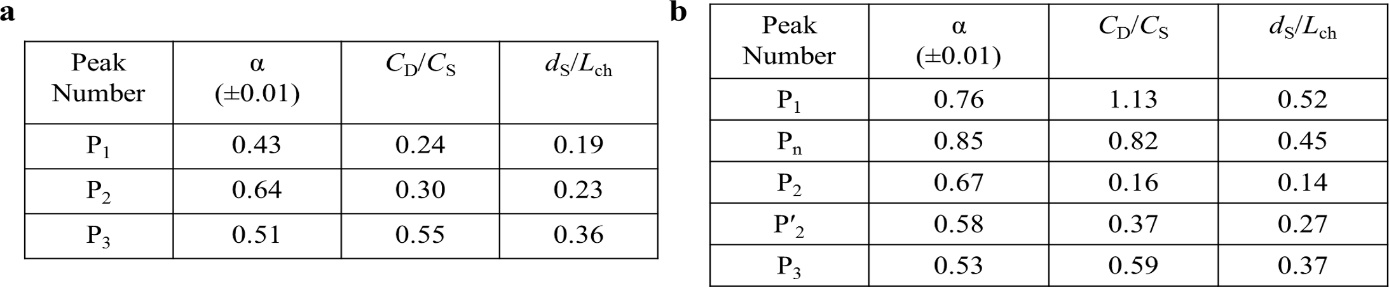


**Supplementary Table S1 ǀ Analysis of current peaks from stability diagrams for device A and B:** (**a)-(b)** Values extracted from the stability diagrams (analysis of the slopes of the Coulomb diamonds) for: lever-arm-factor (α), ratio between drain capacitance and source capacitance (C_D_/C_S_) and relative distance from the QD to source (*d*_S_/*L*_ch_) for each observable current peaks for devices A and device B, respectively. Different current peaks are labeled as different P-donors, consistently with the notations in Figs. S1**a-d**.

In addition, from the *C*_S_/*C*_D_ ratio, which can also be extracted from the analysis of the slopes, we can determine the lateral position of each QD along the channel. This ratio can be converted into the ratio of distances from the QD to the source (*d*_S_) and, respectively, to the drain (*d*_D_). For convenience, we display the lateral position in Tables S1**a** and S1**b** (last column) as *d*_S_/*L*_ch_, where *L*_ch_ is the channel length. This parameter indicates the relative distance of the QD from the source lead (for a QD in the center of the channel, this value would be 0.5). The obtained values suggest that the QDs are located at different lateral positions within the channel. This again suggests that different current peaks originate from different QDs.

Based on this additional quantitative analysis, we suggest that each QD is, in fact, a different P-donor. According to this interpretation, we assign each main current peak (region between Coulomb diamonds) to a different P-donor, as labeled in Figs. S1**a**-**d**, switching its charge states between P^+^ and P^0^. The P-donors are indexed in the order of increasing *V*_FG_. One exception is P_n_ in Figs. S1**b** and S1**d**, which is possibly a P-donor that was not observed in our previous *V*_FG_-*V*_BG_ measurement (Fig. 2**b** in the main manuscript). This new peak is most likely due to the thermal cycling of the device between the two measurements, which induces some changes in the charge distribution in the channel. Only for this P-donor, a small shift between positive and negative polarity of *V*_DS_ can be observed in the stability diagram. The origin of this shift is unclear now, but it does not affect the other observable P-donors.

In our previous work,**^7^** we also evaluated the ionization energy (activation energy) for similar devices from the same sample. This was extracted from Arrhenius plots of the *I*_DS_-*V*_FG_ characteristics measured as a function of temperature. For each isolated current peak, ionization energy was in the range of 20-80 meV. Despite some dispersion, these results are consistent with the ionization energy of P-donors in bulk Si (~45 meV) considering dielectric confinement effect and Coulomb interactions between neighboring P-donors. This further supports our model in which the current peaks are ascribed to individual P-donors.

Recently, it has been shown that the D^-^ state (two-electron occupation of a donor) may also be observed in transport, at least at very low temperatures. Hence, the D^-^ state may be expected to be observed in our devices, too. However, such multiple-electron occupation of the same P-donor is inconsistent with our analysis of stability diagrams, suggesting that D^-^ state cannot be identified in our data. This may be due to the fact that the P-donors in our nanoscale channels are located close to each other and, hence, they are capacitively coupled. Equivalent circuit of this type of possible structure is presented in Fig. S2. Due to this coupling, the charge state of one P-donor could significantly affect the potential of neighboring P-donors. For instance, the first current peak can be ascribed to donor P_1_. However, at slightly higher *V*_FG_, a second donor P_2_ may become available for transport. Once this donor P_2_ captures an electron, it could shift upwards the potential of donor P_1_, too. As a consequence, the D^-^ state of donor P_1_ possibly appears at significantly higher *V*_FG_’s as compared with the case of isolated P-donors. This type of sequence of potential-profile modification with each electron transport through different donors is schematically presented in Fig. S2**a** with the equivalent circuit shown in Fig. S2**b**. Due to such Coulomb interactions, the D^-^ states for each P-donor cannot be easily identified in our devices.

Based on the detailed analysis presented above, it is now clear that the origin of each current peak in the low-*V*_DS_ region in our devices are most likely different individual P-donors, while complex current peaks appearing in the high-*V*_DS_ regions arise due to sequential tunneling through multiple dopants.

**Correlation between shift of the current trace and gate capacitance**

For device B shown in the manuscript, we observe a few sudden changes in the current peak positions (shifts). One of these shifts is more clearly shown in Fig. S3**a**. Based on our model, we ascribe this shift to a sudden change in the gate capacitance of the transport-QD, a change that occurs when two neighboring donor-induced quantum wells merge near the interface. The broader current trace (before the shift) suddenly moves to more negative *V*_FG_ as a thinner trace. In addition, we can observe another thin trace at higher *V*_FG_’s. We ascribe these two traces to consecutive charge states of the merged QD. From the period between two traces (~40 mV), we can evaluate the gate capacitance of the merged QD [*C*_FG_(merged)] to be ~4.0 aF. Since this QD should be about double the size of individual donor-induced quantum wells, we can expect that the gate capacitance for each single-donor QD (*C*_FG_) is about 2.0 aF.


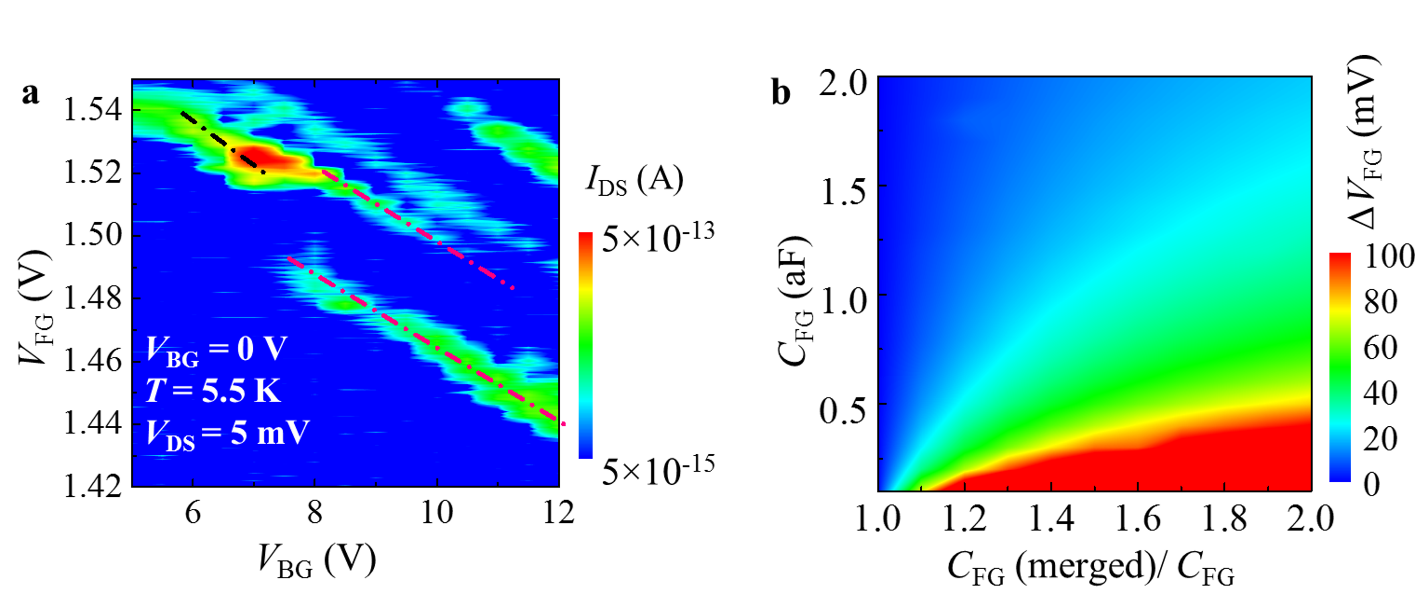


**Supplementary Figure S3 ǀ Correlation between shifts of current trace and gate capacitance.** (**a**) Zoom-in on the first current trace shift region in the *V*_FG_-*V*_BG_ diagram (from Fig. 2b), illustrating the changes that occur before and after the shift. (**b**) Estimation of voltage shift (Δ*V*_FG_) induced by gate capacitance change, monitored in the plane of *C*_FG_-*C*_FG_(merged)/*C*_FG_ (where *C*_FG_ is the (front) gate capacitance of a single donor-induced QD).

In order to confirm the validity of our model quantitatively, we make an analysis of the voltage shift (Δ*V*_FG_) as a function of *C*_FG_ (gate capacitance of the single donor-induced QD) and *C*_FG_(merged) (gate capacitance of the merged QD). In this simulation, we fix the ratio *C*_FG_/*C*_BG_=100 in agreement with our estimation from the experimental results. The simulation results are shown as contour plot in Fig. S3**b**. From this plot, it can be seen that, when *C*_FG_ approaches 2.0 aF and *C*_FG_ (merged) becomes double compared with *C*_FG_, Δ*V*_FG_ is in the range of 20-40 mV (upper-right corner of the diagram). This is consistent with our experimental observation, further supporting our proposed model.

**Example of device with a single current trace shift**

In Fig. 2**b** of the main manuscript, several shifts of current traces to lower *V*_FG_’s can be observed in the high-*V*_BG_ range. This may suggest that all these shifts could be induced by the same positive charge trap. However, we show that such a possibility can be excluded based on the following argument.


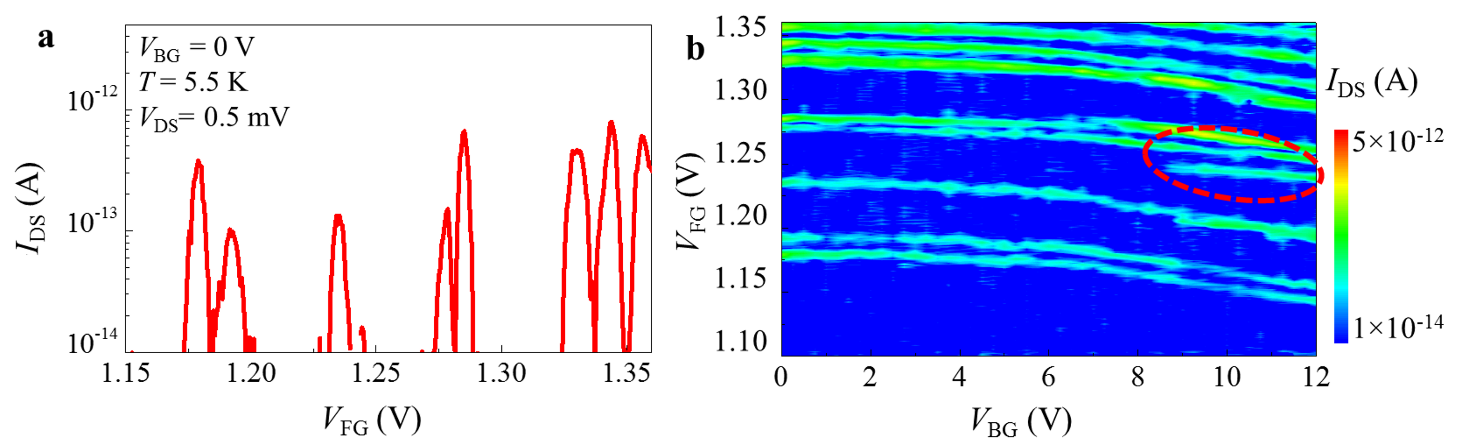


**Supplementary Figure S4 ǀ Diagram for a device exhibiting a single current trace shift.** (**a**) *I*_DS_-*V*_FG_ curve (*T*=5.5 K, *V*_DS_=0.5 mV) at *V*_BG_=0 V for a different device (device C). A number of isolated current peaks, with irregular shapes and no periodicity can also be observed for this device. (**b**) Contour plot of *I*_DS_ as a function of backgate voltage (*V*_BG_) and frontgate voltage (*V*_FG_) measured at *T*=5.5 K. A single current trace shifts to lower *V*_FG_ at *V*_BG_≈9.0 V. Other current traces do not exhibit any sudden shifts in position, smoothly changing as a function of *V*_BG_ and *V*_FG_.

First of all, such a positive charge trap is not expected in our device in which electrons are the charge carriers, as explained already in the main manuscript. Moreover, even in Fig. 2**b** in the manuscript, it can be seen that a few other current traces remain unchanged. A clearer example is introduced here as Fig. S4. This is another device with similar parameters as device A and device B. From Fig. S4**a**, a number of isolated *I*_DS_ peaks with irregular intensities and no periodicity can be observed, similarly to devices A and B. In Fig. S4**b**, however, in the contour plot of *I*_DS_ as a function of *V*_FG_ and *V*_BG_, it can be observed that only a single current trace suddenly shifts to lower *V*_FG_ (as marked), while most other current traces only smoothly change as *V*_BG_ is increased. Such an example clearly suggests that the shifts of the current traces cannot be ascribed to a common external factor, such as a charged trap, but to specific changes in the properties of the QD directly responsible for transport. This interpretation is consistent with the model proposed in our manuscript.

**References:**

1. Sellier, H. et al. Transport spectroscopy of a single dopant in a gated silicon nanowire. *Phys. Rev. Lett.* **97**, 206805 (2006).
2. Prati, E., Belli, M., Cocco, S., Petretto, G. & Fanciulli, M. Adiabatic charge control in a single donor atom transistor. *Appl. Phys. Lett.* **98**, 053109 (2011).
3. Voisin, B. et al. Few-electron edge-state quantum dots in a silicon nanowire field-effect transistor. *Nano Lett*. **14**, 2094-2098 (2014).

4. Tyszka, K. et al., [Comparative study of donor-induced quantum dots in Si nano-channels by single-electron transport characterization and Kelvin probe force microscopy](https://www.researchgate.net/publication/279446492_Comparative_study_of_donor-induced_quantum_dots_in_Si_nano-channels_by_single-electron_transport_characterization_and_Kelvin_probe_force_microscopy?ev=prf_pub). *J. Appl. Phys.* **117**, 244307 (2015).

5. Tyszka, K. et al., [Effect of selective doping on the spatial dispersion of donor-induced quantum dots in Si nanoscale transistors](https://www.researchgate.net/publication/280303776_Effect_of_selective_doping_on_the_spatial_dispersion_of_donor-induced_quantum_dots_in_Si_nanoscale_transistors?ev=prf_pub). *Appl. Phys. Express* 8, 094202 (2015).

6. Waugh, F. R. et al., Single-Electron Charging in Double and Triple Quantum Dots with Tunable Coupling. *Phys. Rev. Lett.* **75**, 705-708 (2011).

7. Hamid, E. et al., Electron-tunneling operation of single-donor-atom transistors at elevated temperatures. *Phys. Rev. B* **87**, 085420 (2013).
